# Differential Producibility Analysis (DPA) of Transcriptomic Data with Metabolic Networks: Deconstructing the Metabolic Response of *M. tuberculosis*

**DOI:** 10.1371/journal.pcbi.1002060

**Published:** 2011-06-30

**Authors:** Bhushan K. Bonde, Dany J. V. Beste, Emma Laing, Andrzej M. Kierzek, Johnjoe McFadden

**Affiliations:** Microbial Sciences Division, Faculty of Health and Medical Sciences, University of Surrey, Guildford, United Kingdom; University of Virginia, United States of America

## Abstract

A general paucity of knowledge about the metabolic state of *Mycobacterium tuberculosis* within the host environment is a major factor impeding development of novel drugs against tuberculosis. Current experimental methods do not allow direct determination of the global metabolic state of a bacterial pathogen in vivo, but the transcriptional activity of all encoded genes has been investigated in numerous microarray studies. We describe a novel algorithm, Differential Producibility Analysis (DPA) that uses a metabolic network to extract metabolic signals from transcriptome data. The method utilizes Flux Balance Analysis (FBA) to identify the set of genes that affect the ability to produce each metabolite in the network. Subsequently, Rank Product Analysis is used to identify those metabolites predicted to be most affected by a transcriptional signal. We first apply DPA to investigate the metabolic response of *E. coli* to both anaerobic growth and inactivation of the FNR global regulator. DPA successfully extracts metabolic signals that correspond to experimental data and provides novel metabolic insights. We next apply DPA to investigate the metabolic response of *M. tuberculosis* to the macrophage environment, human sputum and a range of in vitro environmental perturbations. The analysis revealed a previously unrecognized feature of the response of *M. tuberculosis* to the macrophage environment: a down-regulation of genes influencing metabolites in central metabolism and concomitant up-regulation of genes that influence synthesis of cell wall components and virulence factors. DPA suggests that a significant feature of the response of the tubercle bacillus to the intracellular environment is a channeling of resources towards remodeling of its cell envelope, possibly in preparation for attack by host defenses. DPA may be used to unravel the mechanisms of virulence and persistence of *M. tuberculosis* and other pathogens and may have general application for extracting metabolic signals from other “-omics” data.

## Author Summary

*Mycobacterium tuberculosis* causes tuberculosis, leading to millions of deaths each year. Treatment takes 6 months or more, leading to lack of patient compliance and emergence of drug resistance. The pathogen takes so long to kill because it is able to enter a state of dormancy/latency/persistence where it is insensitive to drugs. There is an urgent unmet need to develop new antibiotics that target dormant/persistent/latent organisms. Most antibiotics target metabolic processes but it is difficult to examine the metabolism of the pathogen directly inside the host or host cells. It is of course possible to identify which genes are active by transcriptomics but there are no established and validated methods to use transcriptome data to predict metabolism. We here describe the development of such a method, called DPA. We validate the method with *E. coli* data and then use DPA to predict the metabolism of the TB pathogen growing inside host cells and from TB sputum samples. DPA demonstrates that the TB bacillus remodels its cells in response to the host environment, possibly to increase the pathogen's defenses against the host immune system. Discovering the metabolic details of this remodeling may identify vulnerable metabolic reactions that may be targeted with new TB drugs.

## Introduction

The *M. tuberculosis* complex includes the human pathogen *M. tuberculosis*, the bovine tubercle bacillus, *M. bovis* and the attenuated vaccine strain derived from *M. bovis*. Tuberculosis (TB) causes 2–3 million deaths each year [1] [2]. Outbreaks of TB due to multidrug-resistant (MDR) and extensively drug-resistant (XDR) strains have become increasingly common in many parts of the world [3]. Control of TB is compromised by the fact that successful treatment takes 6 months or more, leading to lack of patient compliance and subsequent emergence of drug-resistance. These lengthy drug treatment regimes are necessary to kill slowly growing or non-growing cells, known as persisters, in lesions that are refractory to drug treatment [4]. Development of new drugs able to efficiently kill persistent cells could lead to shorter treatment regimes and more effective control of TB. However, very little is known about the physiological and metabolic state of the TB bacillus *in vivo*. The glyoxylate shunt appears to be required during intracellular growth indicating that *M. tuberculosis* survives by scavenging host lipids [5]–[6] [7]; and recent evidence indicates that host cholesterol may be carbon source utilized *in vivo* [8], [9]. Gluconeogenesis has also been shown to be required for growth *in vivo* [10]. There is also growing evidence of a shift to anaerobic respiration during dormant/latent/persistent infection [11] [12] [13]. These findings have been useful in directing rational drug development [14] but a more complete understanding of *M. tuberculosis* metabolism *in vivo* remains a major goal of TB drug research.

There are many approaches to studying the physiology of bacterial cells *in vitro*. High throughput methods such as metabolomics, proteomics and transcriptomics may be combined with traditional biochemical, physiological and structural investigations to define the physiological state of the organism *in vitro*. These data may be incorporated into genome-scale models of bacterial cells to build virtual cells capable of simulating the growth of bacteria [15] [16] [17] both *in vitro* and *in vivo*. Recently, two genome-scale models of the TB bacillus have been published [18] [19] allowing this approach to be applied to modeling the physiological state of the TB bacillus during infection. However, whereas it is relatively straightforward to obtain multiple measurements for bacteria to define a physiological or metabolic state *in vitro*, only limited information can be obtained for *in vivo*. In particular, it is very challenging to perform metabolomic, proteomic, biochemical, physiological or structural studies with the small numbers of organisms obtained from infected tissue. However, it is possible to perform transcriptome studies on *in vivo* grown organisms and these methods have been applied to the TB bacillus to obtain transcriptome profiles of bacteria growing in cultured macrophages, mouse models and in human lesions [20], [21] [22] [23] [24]. The transcriptional profile of a cell can define most aspects of its physiological state; therefore it should be possible to predict a physiological state from knowledge of its complete transcriptome. However the mapping between messenger RNA levels and physiological state is highly complex and non-linear depending on many unknown factors such as mRNA stability, translation efficiency and post-translational modification of proteins. Traditional approaches to defining metabolic responses from transcriptome data have generally relied on examining expression levels of key (rate-controlling) genes in metabolic pathways (for instance, [25]. However, metabolic control analysis has demonstrated that control is distributed throughout the entire metabolic network, such that the flux through any particular pathway is controlled globally [26], [27] rather than by a particular enzymatic step. This makes a simple mapping of differentially expressed genes onto metabolic pathways an unrealistic strategy for successful predictions of global metabolic state changes.

Several system-level approaches have been proposed to extract metabolic information from gene expression profiles. In the reporter metabolites approach [28] the local connectivity of a metabolite in the bi-partite, substance/reaction graph is used to identify a set of genes associated with each metabolite. Subsequently, for each of the metabolites, the distribution of the microarray-derived signal of genes associated with the metabolite is compared with the background distribution of the microarray-derived signal for all genes, resulting in the identification of the transcription regulation focal points of metabolism: network nodes that are directly affected by clusters of differentially expressed genes. In another approach, Shlomi [29] used Mixed Integer Linear programming to minimize the discrepancy between the internal metabolic flux distribution and the transcriptional profile of genes encoding metabolic enzymes. Their approach identifies flux distributions, which are consistent with the stoichiometric constraints of the genome scale metabolic reaction network and at the same time maximize the number of active metabolic fluxes associated with up-regulated genes and the number of non-active metabolic fluxes associated with down-regulated genes. Yet another approach, E-flux, was recently developed and used to examine *M. tuberculosis* microarray data in the context of the genome scale metabolic reaction network, by constraining upper bounds of metabolic reactions to values proportional to the microarray signals of genes associated with these reactions [30]. In this study we describe a novel approach, Differential Producibility Analysis (DPA), which uses FBA to analyze microarray data in the context of a genome scale metabolic network. The DPA method differs from both the Shlomi method [29] and the e-flux method [30] in avoiding assumptions concerning the relationship between transcriptional signal and metabolic flux, depending instead (like the reporter metabolite approach) only on network structure. However, by application of FBA to associate metabolites with genes, rather than graph theoretical approaches, the DPA method is more global than the reporter metabolite approach [28] and examines metabolites in the context of the entire metabolic network rather than their local network environment. Each of these approaches does however have its merits and data are not yet available to identify the optimal means of predicting metabolism from transcriptional profiles.

To test and validate the DPA approach we first applied it to a transcriptional dataset obtained from a well-characterized system so that the results of DPA could be compared with a known biological response. We chose to analyze the transcriptional response of the enteric bacterium *E. coli* to reduced oxygen availability and the impact of the global regulator, FNR, on that response [25]. Having validated the method we then applied DPA to analyze transcriptomic data in the context of one of the available network models of *M. tuberculosis* [18]. Our aim was to utilize differential transcript data obtained for a number of *in vitro* and *in vivo* states to identify characteristic global metabolic changes. We focused our studies on attempting to define metabolic changes associated with the adaptation of *M. tuberculosis* to the *in vivo* environment, as represented by macrophage-grown *M. tuberculosis* and human sputum-derived *M. tuberculosis*. We compared metabolic profiles for adaptation to the *in vivo* environment to adaptation of *M. tuberculosis* to a range of *in vitro* environments in an attempt to deconstruct the *in vivo* state into components that can be studied *in vitro*.

Our analysis revealed a previously unrecognized feature of the response of *M. tuberculosis* to the macrophage environment: a down-regulation of genes influencing metabolites in central metabolism. This was accompanied by up-regulation of many genes that influence synthesis of cell wall components and virulence factors, which had been identified in the original transcriptome study [22]. The results suggest that a significant feature of the response of the tubercle bacillus to the intracellular environment is a channeling of resources towards remodeling of its cell envelope, possibly in preparation for attack by host defenses system. The study demonstrates that the DPA method can successfully extract metabolic signals from transcriptomic data and can be used to study global effects of gene regulatory changes. The method may have general application for extracting metabolic signals from other high-throughput “–omics” data.

## Results

### Differential Producibility Analysis (DPA)

**Figure 1:**
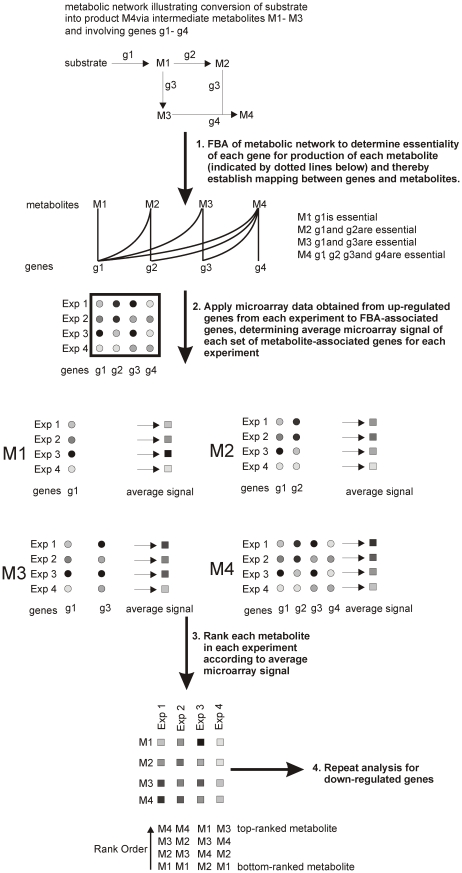
Schematics of DPA illustrated through analysis of a toy metabolic network consisting of 4 metabolites (M1–M4) and 4 genes (g1–g4).

The aim of DPA is to identify those metabolites that are likely to be most affected by a system-wide change in gene transcription. Essentially DPA links up- or down-regulated genes with metabolites, using FBA essentiality, rather that more traditional pathway identification tools (such as KEGG) or network analysis. Because a large number of genes in multiple pathways of metabolism can be essential to produce any particular metabolite, DPA captures a more system-wide association between genes and metabolites than are captured by any simple pathway approach. Figure 1 illustrates the principle of the method for a toy example metabolic network consisting of four metabolites and four genes. To perform DPA we first utilized a FBA-based metabolite producibility plot [31] to identify, at a system-level, the sets of genes that participate in the production of each metabolite (Figure 1, step 1). Subsequently, for each of the metabolites, we calculated a metabolite signal, defined as the median microarray-derived data signal for those genes that affect its production (Figure 1, step 2). Therefore, for each microarray dataset, representing the experimental condition of interest, we generated a vector of metabolite signals. To avoid negation of gene expression signals for metabolites which were associated with different sets of up- and down-regulated genes, the analysis was performed separately for up and down-regulated genes. Each metabolite in the network was then ranked according to the average intensity of microarray signal associated with genes that affect its production (Figure 1, step 3) using the non-parametric Rank Products Analysis [32], which has been shown to be the method of choice for meta-analysis of microarray datasets derived by different research groups on different experimental platforms. The detailed steps are described in the Methodology section. The resulting ranked lists of metabolites that were identified by DPA to be most affected by the transcriptional response (we term these the *affected metabolites*) for all the conditions were then subjected to cluster analysis to identify similar experimental conditions that result in similar changes in global metabolic state and to identify metabolites that have common metabolite signal profiles across these experimental conditions examined.

### DPA analysis of *E. coli* microarray datasets

To investigate the utility of DPA for extracting metabolic signals from transcription data we performed an analysis of microarray data obtained from an experiment which aimed to identify the transcriptional response of the enteric bacterium *E. coli* to reduced oxygen availability and the impact of the global regulator, FNR, on that response [25]. *E. coli* is a facultative anaerobe that can survive and replicate in both aerobic and anaerobic environments. Although preferentially utilizing aerobic respiration, the organism is able to respond to reduced oxygen availability by shifting its metabolic pathway utilization to mixed acid fermentation and anaerobic respiration. The two-component global regulator, FNR, is known to be involved in regulating the shift between aerobic and anaerobic metabolism. FNR is a CAP (catabolite activator protein) homologue that contains an oxygen labile iron-sulfur centre, which acts as the oxygen sensor, and was known to regulate over 70 different genes. The aim of the target study was to identify additional genes that are differentially expressed in response to oxygen availability and discover which global changes were mediated by FNR. The study was designed to measure the transcriptomic profile of wild type (strain MC4100) *E. coli* grown under aerobic (+O_2_) and anaerobic (−O_2_) conditions and also FNR- strain (MC4100 Δ*fnr-2*) of *E. coli* grown only under anaerobic conditions in order to identify the FNR response, Note that the original paper classified genes into eight different regulatory patterns (I to VIII) depending on whether gene expression was higher, lower or unchanged in response to oxygen and whether or not the response was FNR-dependent.

We performed DPA analysis of the two paired conditions: +O_2_/−O_2_ and (−O_2_)+FNR/−FNR in the context of a genome-scale metabolic model of *E. coli* [33]. We obtained four lists: Table S1a, metabolites identified as associated with down-regulated genes on exposure of wild-type *E. coli* to anaerobic conditions (corresponding to regulatory patterns II, IV, V of [25]), Table S1b, metabolites associated with up-regulated genes in the wild-type in response to anaerobic growth (regulatory patterns I, III, VII), Table S1c, metabolites associated with increased gene expression in the FNR- strain grown under anaerobic conditions (regulatory patterns I, V, VI), and Table S1d, metabolites associated with decreased gene expression in the FNR-strain grown under anaerobic conditions (regulatory patterns II, VII, VIII). These lists are presented in Supplementary Tables S1a–d with brief annotations of the most-significantly associated metabolites indicating predominant metabolic pathways associated with each metabolite. We compared DPA analysis with gene identification and ontology analysis, as performed in the original published text [25].

### Metabolites identified by DPA to be associated with down-regulated genes on exposure of wild-type *E. coli* to anaerobic conditions (and thereby up-regulated during aerobic growth)

The most notable characteristic of this metabolite list (Table S1a) was the inclusion of many sugars (*e.g.* glucose and fructose) and carbohydrates (*e.g.* glycogen) as well as intermediates (e.g. glyceraldehyde-3-phosphate and dihydroxyacetone phosphate) that are metabolized predominantly aerobically via glycolysis and the TCA cycle (*e.g.* fumarate). The microarray analysis similarly identified genes involved in sugar metabolism (*e.g.* three genes of the *manXYZ* operon and *ptsG* glucose phosphotransferase system II) and the TCA cycle (*e.g.* genes encoding several components of pyruvate dehydrogenase and alphaketogluterate dehydrogenase) as being down-regulated during the shift to anaerobic growth. Metabolic signals that were additionally identified by DPA include iron (an essential cofactor of cytochrome oxidase, whose genes were found to be repressed during anerobic growth) and several metabolites involved in the pentose phosphate cycle and propionate metabolism (*e.g.* the methylcitrate cycle) as well as several metabolites involved in lipopolysaccharide synthesis.

### Metabolites identified by DPA to be associated with up-regulated genes on exposure of wild-type *E. coli* to anaerobic conditions

Under anoxic conditions, the active form of FNR exists as a dimer with each monomer bound to an iron-sulfur cluster. The sulfur within the cluster is donated by cysteine (through the action of cysteine desulfurase) but glutathione is required to maintain the complex in the reduced state [34]. It is thereby notable that both cysteine and glutathione are identified by DPA amongst the metabolites associated with the most up-regulated genes during anaerobic growth (Table S1b); a response that was not detected in the analysis of microarray signals. Hydrogen sulfide, both a substrate of cysteine synthase and product of anaerobic respiration, was also identified in this group. Consistent with the microarray signal was the DPA identification of chloride as a metabolite associated with up-regulated genes during anaerobic growth (three genes encoding a homologue of the mammalian *yadQ* chloride channel were identified as up-regulated [25]). More puzzling was the DPA identification of many metabolites involved in peptidoglycan and glycerolipid synthesis. A few genes involved in lipid synthesis, such as acetyl-coA transferase, were identified in the microarray signal and a novel anaerobic β-oxidation pathway has recently been identified to be active during anaerobic growth of *E. coli* [35].

### Metabolites associated with increased genes expression in the FNR- strain grown under anaerobic conditions (and thereby repressed by FNR in the wild-type)

Many of the metabolites identified in this group (Table S1c) were also those identified by DPA ((i) above)) as being associated with down-regulated genes on exposure of wild-type *E. coli* to anaerobic conditions (and thereby up-regulated during aerobic growth), consistent with the involvement of FNR in repressing aerobic pathways.

### Metabolites associated with decreased gene expression in the FNR-strain grown under anaerobic conditions (and thereby induced by FNR during anaerobic growth in the wild-type)

The most notable characteristic of this list (Table S1d) was the inclusion of several fermentation products including ethanol, formate and acetaldehyde, consistent with a metabolic shift of *E. coli* towards mixed acid fermentation under anaerobic conditions. This signal was detected in the microarray analysis (*e.g.* both formate and acetaldehyde dehydrogenase genes demonstrated FNR-dependent induction in anaerobic growth). Also notable is cobalamin, cofactor of the B12-dependent nucleotide reductase. This signal was not identified in the original microarray study but transcription of the *nrdDG* operon, which encodes this class III nucleotide reductase, has since been shown to be strongly induced by anaerobiosis in a FNR-dependent manner [36].

### DPA analysis Of *M. tuberculosis* microarray datasets

Our principal aim was to gain insight into the *in vivo* metabolic state of *M. tuberculosis* by comparison with growth in various *in vitro* conditions (Table 1). We therefore performed DPA analysis of microarray datasets in the context of the GSMT-TB metabolic network of *M. tuberculosis* [18]. The first microarray dataset we examined was taken from a study of *M. tuberculosis* replicating in mouse macrophage [22]. The second model of the *in vivo* state was a study on *M. tuberculosis* cells isolated from human sputum of patients with TB [37]. The *in vitro* datasets we examined were from a number of studies that performed microarray analysis of *M. tuberculosis* growing on different substrates (succinate, palmitate), or exposed to various toxic conditions (treatment with hydrogen peroxide, low pH, UV radiation), or growth-limited in conditions that are thought to mimic aspects of growth limitation *in vivo* (NRP1 cells in the microaerobic ‘Wayne model’ of dormancy [38], slow growth in the carbon-limited chemostat [39]). Note that this is an *in vitro* model of dormancy/persistence and its relationship to the asymptomatic and un-infectious state (often known as latency but sometimes also referred to as dormancy or persistence) that is encountered *in vivo* remains unclear [4]. DPA analysis of all datasets is shown in the supporting information (Figure S1). The source of each dataset is detailed in Table 1. The mouse macrophage study examined *M. tuberculosis* grown in macrophages that were either naïve or activated, wild-type or *Nos1* mutants, at various time-points. The experiments utilized two different microarray formats. DPA was performed on each dataset. As can be seen by (Figure S1), the DPA results, most of the mouse macrophage experiments clustered closely together so for most subsequent studies, the result of only one typical experiment (48 hr infection, activated macrophages, amplicon microarray probes) is discussed further. One-class Rank Products analysis was performed (see Materials and Methods) to identify the top 100 metabolites predicted by DPA to be associated with up-regulated genes and down-regulated genes (Supporting information, Table S2) for each dataset.

**Table 1:** Abbreviations and source of *M. tuberculosis* transcriptome data used in this study.

No	Abbreviation	Experiment	Reference
1	Macrophage	Activated (48 hr) macrophage	[22]
2	Peroxide.1	H2O2 treatment	[22]
3	Palmitate.1	PA treatment	[22]
4	Peroxide.2	H2O2 treatment	[58]
5	Azide	NaN3 treatment	[58]
6	NRP1exp	NRP-1 (oxygen-limited)	[58]
7	Palmitate.2	Growth on palmitate	[58]
8	Ph4.8	pH 4.8 (acidic) treatment	[58]
9	Succinate	Growth on succinate	[58]
10	UV	Growth under UV radiation	[58]
11	Chemostat	TB growth under chemostat	[44]
12	Sputum	Sputum sample analysis from TB patients	[37]

### The metabolic response associated with adaptation of *M. tuberculosis* to growth in macrophages

**Figure 2:**
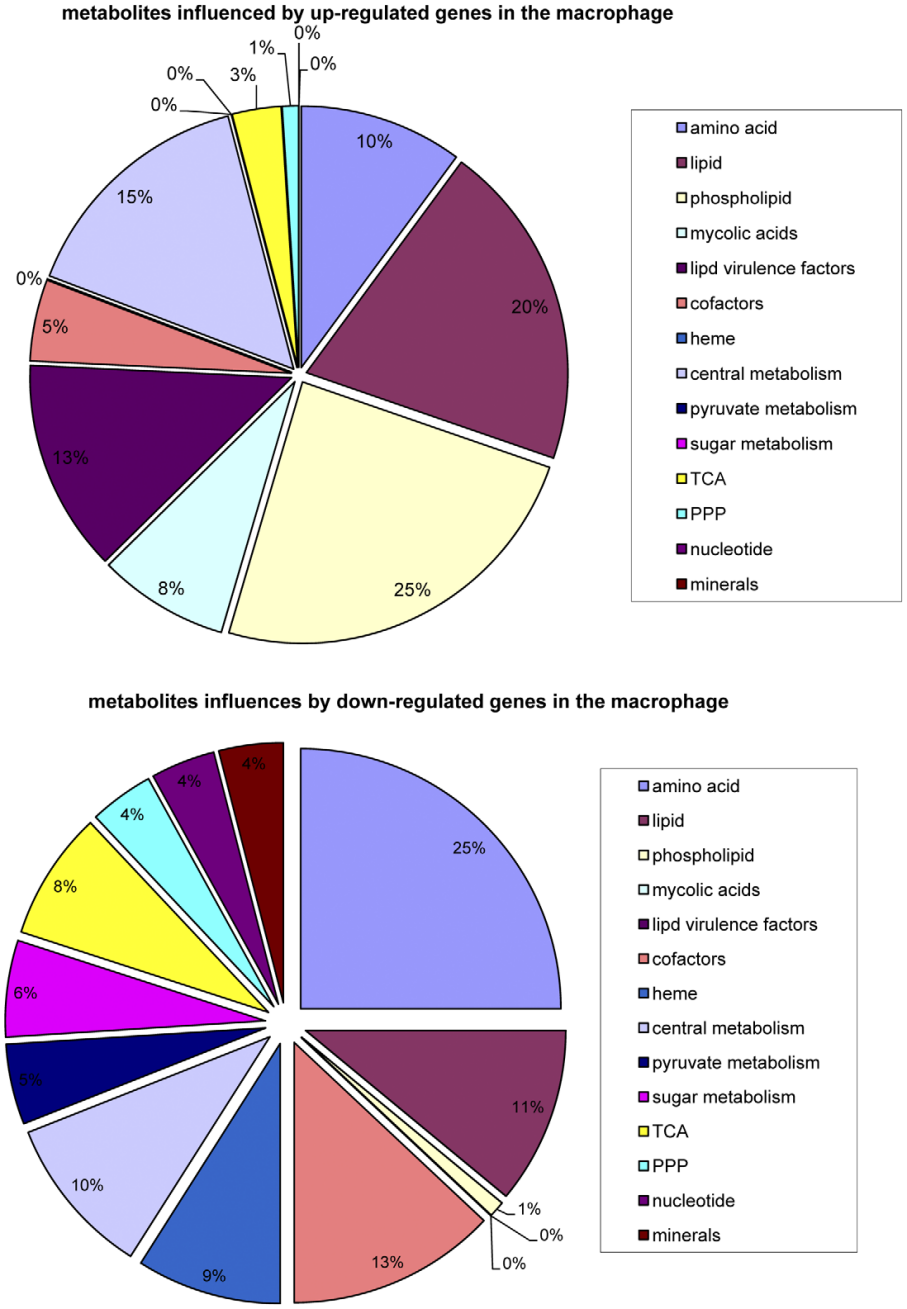
Pi chart illustrating the role of *M. tuberculosis* metabolites in macrophages. Pi chart illustrating the role of metabolites associated by DPA with up-regulated (A) or down-regulated (B) genes in the mouse macrophage.

Each of the top 100 metabolites associated by DPA with either up- or down-regulated genes in the macrophage was classified into broad areas of metabolism (*e.g.* nucleotide or lipid biosynthesis) based on the pathway(s) that involved this metabolite in the GSMT-TB model [18]. The full list of metabolite assignments can be seen in Table S5. A pie chart was then used to compare the metabolic processes most affected by up (Figure 2A) or down-regulated (Figure 2B) *M. tuberculosis* genes when it adapts to the mouse macrophage. As can be seen, there are large differences. The most striking are: (i) metabolites involved in phospholipid synthesis (principally phosphatidylinositol mannoside (PIM)), mycolic acid synthesis, cell wall virulence factors (phenolphthiocerol dimycocerosate (PDIM), phenolic glycolipid (PGL), acyltrehalose, mannosyl beta-1-phosphodolichol (MPD)) are mostly associated with up-regulation in the macrophage; (ii) metabolites associated with various central metabolism pathways were mostly associated with down-regulation in the macrophage. This was particularly apparent for metabolites involved in amino acid metabolism that comprised 25% of the top 100 metabolites in the down-regulated list but only 10% of metabolites in the up-regulated. Other central metabolic themes that were mostly associated with down-regulation in the macrophage were TCA cycle metabolites, sugar metabolism, pyruvate metabolism (5% compared to zero), cofactors (13% compared to 5%, the cofactors involved were mostly involved in biotin, thiamine and F420 synthesis), nucleotide synthesis (4% compared to 0%) and heme synthesis (9% compared to 0%). Also noteworthy, although the numbers are small, is the observation that several metabolites involved in anaerobic respiration (e.g. nitric oxide and molybdenum, which is a co-factor for nitrate reductase) were associated with down-regulated genes.

### The metabolic response of *M. tuberculosis* isolated from human sputum

To identify those metabolites that were most affected by the transcriptional response of *M. tuberculosis* to the human sputum environment a similar analysis to that documented above was performed. The highest ranked metabolites associated with up- and down-regulated genes in *M. tuberculosis* isolated from human sputum (Supporting information, Table S2) were, perhaps surprisingly, not particularly closely related to metabolites associated with adaptation to the macrophage environment. Metabolites associated with up-regulated genes were associated with many cell wall components and virulence factors, such as metabolites involved in peptidoglycan synthesis, synthesis of the sulfolipid virulence factor SL-1 [40] (which was not up-regulated in mouse macrophages), synthesis of arabinogalactan [41], synthesis of phenolphthiocerol dimycocerosate (PDIM) [42], synthesis of phosphatidylinositol mannoside (PIM) [43] and *de novo* biosynthesis of nucleotides (e.g. deoxyuridine-diphosphate, DUDP and deoxycytidine monophosphate, DCMP). Metabolites associated with down-regulated genes in the sputum-derived cells included several metabolites involved in amino acid synthesis but also synthesis of several cofactors such as molybdenum and coenzyme A (e.g. L-pantoate, PANT). Oxygen was also identified as associated with down-regulated genes indicating perhaps a down-regulation of genes involved in aerobic respiration.

### The metabolic response of *M. tuberculosis* to various *in vitro* conditions

The lists of top-ranking metabolites associated by DPA with each *in vitro* condition (Supporting information, Table S2) showed many interesting features. For instance, exposure to pH 4.8 was associated with up-regulation metabolites such as 2-C-methyl-D-erythritol-2,4-cyclodiphosphate (MDECPP) involved in polyprenoid synthesis and metabolites involved in arabinogalactan synthesis, but down-regulation genes were associated with metabolites (such as 3-dehydroquinate, DQT) involved in aromatic amino acid synthesis. Genes that were up-regulated on exposure to hydrogen peroxide were associated with metabolites involved in synthesis of the secreted siderophore mycobactin and cobalamin synthesis but metabolites associated with down-regulated genes were involved in peptidoglycan, nucleotide and amino acid. Genes that were upregulated by slow growth of *M. tuberculosis* in the chemostat were associated with metabolites such as 2-methyl citrate and α-ketoglutarate, involved in the methyl citrate cycle and TCA cycle respectively, together with several metabolites involved in cobalamin synthesis (e.g. hydrogenobyrinate), biotin synthesis (e.g. 8-amino-7-oxononanoate), heme synthesis (e.g. protoporphyrin-IX) and glutamate synthesis (e.g. N-acetyl-L-glutamate). Genes that were up-regulated in the Wayne model of dormancy NRP1 state [38] were associated with many mycolic acid synthesis intermediates.

### Comparison between datasets: deconstructing the macrophage response

**Figure 3:**
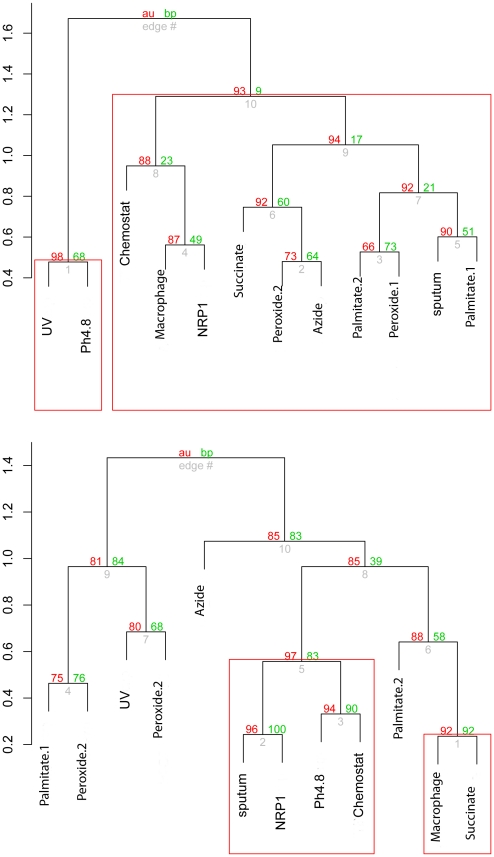
Clustering of *M. tuberculosis* gene expression datasets. Clustering of gene expression datasets based on DPA of metabolites associated with up-regulated genes, A) and down-regulated genes, B). Red numbers indicate the Approximately Unbiased (au) multiscale bootstrap based *p*-value for dendrogram. We used 90% confidence to identify significant clusters (with Red bounding boxes).

To gain insight into the metabolic overlap between the macrophage-grown *M. tuberculosis* and various *in vitro* model systems, we performed hierarchical cluster analysis of the resulting ranked metabolite lists for all the experimental conditions followed by identification of common metabolites in various experimental conditions using Venn diagrams. First, metabolite signals (median gene expression value for each metabolite) were ranked for each experimental datasets. The experiments were then subjected to hierarchical clustering using Pearson's correlation coefficient of metabolite signal ranks as a similarity measure. The trees reflecting the degree of correlation between metabolite ranking order in each experiment are shown on Figures 3a and 3b. Statistically significant tree nodes, conserved in over 90% of bootstrap replicates were identified as clusters. The analysis of metabolites affected by up-regulated genes (Figure 3a) groups the NRP1 Wayne model of dormancy experiment [38], the slow-growth chemostat model [18] and the macrophage derived *M. tuberculosis* into a cluster separated from the remaining part of the tree at the node conserved in 92% of bootstrap replicates. The close match between the macrophage experiment and the slow growth in the chemostat experiment suggests that *M. tuberculosis* is similarly growth restricted in the macrophage environment. Slow growth is also a component of the NRP1 cells, which may account for the inclusion of this experiment into the same cluster. The cluster analysis of metabolites associated with down-regulated genes reveals a statistically significant cluster of four conditions containing NRP1 cells, sputum, the slow-growth chemostat model and *in vitro* acid stress. It is interesting to note that for metabolites associated with both up and down-regulated genes the *in vivo* model conditions (human sputum and mouse macrophage derived *M. tuberculosis*) do not cluster together suggesting that different metabolic pathways are activated in *M. tuberculosis* residing in mouse macrophages compared to human sputum.

**Figure 4:**
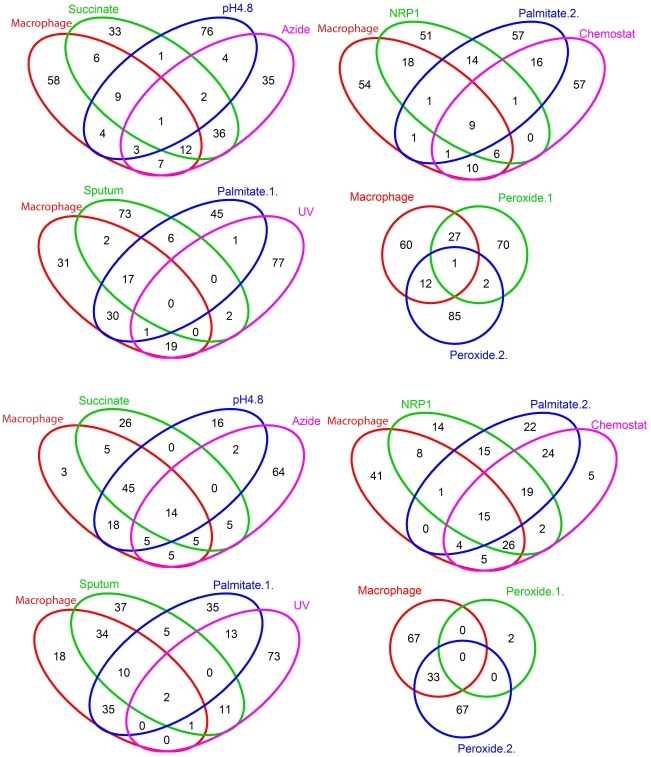
Venn diagrams comparing *M. tuberculosis* metabolites associated by DPA with different experimental conditions. Venn diagrams comparing the top 100 significant metabolites associated with up-regulated (A) and down regulated (B) genes (refer to Supplementary Information Table S2 for the detailed metabolite list for each experimental condition).

To identify specific metabolites responsible for the observed level of clustering, we used Venn diagrams to compare the 100 top ranked metabolites associated with up- and down-regulated genes for each condition. The Venn diagram analysis, presented in Figure 4, was performed in groups of three conditions that were compared with each other and the *M. tuberculosis* macrophage experiment (except for the last comparison which was with only two conditions compared to the macrophage experiment). Each experimental condition was associated with metabolites unique to that condition and metabolites that were shared associated with a range of conditions, as detailed in Supplementary Tables (Tables S3 and S4 for metabolites associated with up- and down-regulated genes respectively). Several interesting features of the analysis are apparent. Firstly, metabolites associated with up-regulated genes from macrophage-grown *M. tuberculosis* (Table S3) contained only one metabolite unique to the macrophage (a mycolic acid intermediate). No unique metabolites were associated with down-regulated genes in the mouse macrophage (Table S4). Many metabolites could be identified that were shared between the macrophage experiment and other experiments. Some were shared with very few other conditions whereas others were found in several *in vitro* experiments. For example, metabolites involved in synthesis of the virulence-associated phosphatidylinositol mannoside (PIM) cell wall phospholipid were associated with up-regulated genes in the macrophage and growth on palmitate (palmitate.1. Note that the analysis includes two pairs of conditions – peroxide and palmitate growth – that were commonly examined in the two different studies. Perhaps surprisingly, the metabolite signal was not very similar for these paired conditions. This probably reflects differences in the way the experiments were performed in the different laboratories). Metabolites involved in synthesis of the virulence determinant, phenolphthiocerol dimycocerosate (PDIM), were associated with up-regulated genes in the macrophage but also the *in vitro* conditions NRP1, growth on palmitate (palmitate.2) and exposure to either pH 4.8 or sodium azide. Metabolites shared between up-regulated genes in both *M. tuberculosis* grown in macrophages and *M. tuberculosis* isolated from human sputum included several 3-carbon metabolites, such as D-glyceraldehyde-3-phosphate and monoacylglycerol, both involved in triglyceride synthesis. Metabolites involved in cholesterol metabolism were shared between macrophage-grown *M. tuberculosis* and growth on palmitate, which is consistent with cholesterol and palmitate requiring similar lipid oxidation pathways for catabolism. More surprisingly, metabolites involved in cholesterol metabolism were also associated up up-regulated genes during slow growth in the chemostat and NRP1 in the Wayne model of dormancy, despite the fact that cholesterol was not available in either condition. The result may indicate that pathways involved in cholesterol metabolism may be activated in *M. tuberculosis* by non-lipid signals, such as nutrient starvation.

As already mentioned, there were no unique metabolites associated down-regulated genes in the macrophage (Table S4). Most of the metabolites common to the macrophage and *in vitro* conditions were involved in central metabolism, amino acid synthesis/degradation, and respiration. However, some specific responses were also apparent. Metabolites (e.g. protoporphyrin-IX) involved in heme synthesis were commonly associated with down-regulated genes in macrophages, growth on succinate, and exposure to pH 4.8. Pentose phosphate pathway metabolites, such as ribose and deoxyribose-5-phosphate (DR5P) were commonly associated with down-regulated genes in the macrophage, NRP1 and slow growth in the chemostat, as were several metabolites involved in amino acid synthesis, such as 3-hydroxy-isobutyrate (HIBUT) and 3-phospho-hydroxypyruvate (PHP). Metabolites involved in biotin synthesis were associated with down-regulated genes in macrophages and growth on palmitate (palmitate.1).

## Discussion

Several different methods are currently available for extracting metabolic information from transcriptome data. The method that has been used the longest is simply to infer metabolic changes from the nature of genes that are up or down-regulated. However, one of the key insights of systems biology is that control of any system tends to be distributed so the activity of any single gene does not necessarily reflect the state of the system as a whole. For this reason, recent attempts to extract metabolic information from transcriptome data have utilized genome-scale metabolic models as a tool to extract system-level metabolic signals. DPA is such a system-based method that analyzes transcriptome data on a framework of gene-metabolite relationships established by FBA. It is a global method that examines the contribution of every gene in the network to the production of every metabolite in the network.

There are several aspects of DPA that merit consideration. First, unlike the method of Shlomi [29] and the E-flux method [30] it makes no attempt to predict actual fluxes. We believe that such an approach is realistic, given the uncertainties of the mapping from the transcriptome through the proteome to the metabolome; and we note that flux predictions of the above methods had not yet been checked against experimentally-derived fluxes, such as those obtained by 13C-Metabolic Flux Analysis. DPA can be compared to the reporter metabolite approach [28], but, rather than relying on local connectivity, DPA utilises FBA to establish genome-scale linkages between metabolites and genes and may thereby detect the influence of distant gene expression events on each metabolic step. To put it another way, whereas the reporter metabolite method ‘is basically a test for the null hypothesis, “neighbour enzymes required for synthesis of a metabolite in the metabolic graph show the observed normalized transcriptional response by chance”’ [28]; DPA is a test of the null hypothesis, “all enzymes required for synthesis of a metabolite in the metabolic graph show the observed normalized transcriptional response by chance.” Secondly, DPA separately analyses metabolites associated into both up- and down-regulated genes. This does generate some anomalies, such as the occasional presence of the same metabolite in both sets of lists (a metabolite associated with both up- and down-regulated genes – but usually with very different rank order), but its advantage is that it avoids cancellation of signal from metabolites associated with both up- and down-regulated genes. Secondly, we found that a few metabolites were not examined by DPA. This was found to be due to redundancy in synthesis pathways, rendering some metabolites (often in central metabolic pathways) essentially invisible to differential producibility analysis since no genes were essential for their production. Nevertheless, despite these potential drawbacks, DPA proved to a powerful tool for extracting metabolic signals from transcriptome data.

We chose to test DPA using an established dataset obtained from a well-characterized system: the response of *E. coli* to anaerobic growth and the role of the FNR global regulator [25] in that response. The value of DPA was clearly demonstrated by its consistency with gene ontology identification in the original study of a metabolic shift from aerobic sugar utilization via glycolysis, pentose phosphate pathway and TCA cycle towards anaerobic utilization of fermentation pathways [25]. DPA also identified repression of the aerobic pathways for sugar utilization as a major target for the action of FNR. However, DPA was able to identify additional metabolic signals in the transcriptome data that was not identified by gene ontology analysis. For instance, DPA identified components of cysteine and glutathione metabolism as being activated during anaerobic growth which is consistent role of these metabolites in activation of FNR under anaerobic conditions [34]. Also, cobalamin synthesis was highlighted by DPA as being FNR-dependent and although this had not identified in the original study, the role of FNR in cobalamin synthesis, has since been demonstrated [36].

With the value of DPA demonstrated in a well-characterized system, we then used the technique to investigate the transcriptional response of *M. tuberculosis* to environmental stresses and replication in the macrophage. Firstly, we were able to identify previously recognized features in several of the datasets we examined, confirming the utility of the method. For instance, Schnappinger [22] identified genes (such as *UmaA*) involved in mycolic acid synthesis that were up-regulated in *M. tuberculosis* grown inside macrophages, and we were also able to identify several intermediates in mycolic acid synthesis that were associated with up-regulated genes in the same dataset. Similarly, Beste [44] identified protein synthesis and modification as being down-regulated in slow-growing *M. tuberculosis* in the chemostat, and metabolites associated with amino acid metabolism were found to be associated with down-regulated genes in the same dataset (a response shared with macrophage and NRP1-derived cells) by DPA analysis. DPA also revealed features of exposure of *M. tuberculosis* to *in vitro* environments that were consistent with existing knowledge. For instance, genes that were found to be up-regulated in both macrophage-grown *M. tuberculosis* and *M. tuberculosis* exposed to hydrogen peroxide included several metabolites, such as mycobactin, involved in iron uptake. Iron is a cofactor of the enzyme catalase that would be expected to be up-regulated on exposure to peroxide. Increased catalase synthesis would require increased iron uptake and therefore increased synthesis of the siderophore mycobactin. That this response is common between mouse macrophage-grown *M. tuberculosis* and peroxide-treated *M. tuberculosis* is consistent with data indicating that *M. tuberculosis* is exposed to oxidative stress in the mouse macrophage [45]. DPA thereby allows the response of *M. tuberculosis* to the host environment to be deconstructed into components that can be studies *in vitro*.

One of the most striking features of DPA analysis of the macrophage data was the number of metabolites associated with up-regulated genes that are involved in the synthesis of cell envelope lipid components including mycolic acids and envelope-associated several virulence factors such as phosphoinositol mannosides and phenolic glycolipid are associated with upregulated genes when *M. tuberculosis* is growing in macrophage metabolites. The most likely explanation is that exposure to the macrophage environment stimulates *M. tuberculosis* to remodel its outer surface, as was suggested from analysis of the microarray data [22], possibly in preparation for attack by host defenses. A distinctive feature of the TB bacillus is its high lipid content (approximately 40% of its cell mass) and the large number of genes (more than 250) involved in lipid and polyketide synthesis and degradation in the TB genome (Cole *et al.*, 1998). Several antituberculous drugs, such as isoniazid and pyrazinamide, are known to target lipid biosynthesis. It is widely believed that a key component of the adaptation of the TB bacillus to the intracellular environment is a switch to consuming host lipids [5] including activation of genes, such as the isocitrate lyase gene, involved in the degradation of lipids. However, our analysis indicates that lipid metabolism in *M. tuberculosis* during infection is also likely to be directed towards lipid biosynthesis involved in remodelling the cell surface. Unraveling the molecular details of this remodeling may provide novel drug targets.

However, in addition to areas of metabolism that were highlighted in the original macrophage transcriptome study, DPA identified several other components of the adaptation of *M. tuberculosis* to the macrophage that had not been identified in the original analysis, such as cofactor synthesis. Genes involved in the synthesis of the cofactors cobalamin and F420 tended to be up-regulated in the macrophage whereas genes associated with synthesis of biotin tended to be down-regulated. Coenzyme F420 is involved in redox reactions such as the F420-dependent glucose-6-phosphate dehydrogenase and is required for activation of the experimental antituberculosis drug PA-824 by *M. tuberculosis* [46]; whereas cobalamin is involved in several biosynthetic reactions including nucleotide and fatty acid synthesis and has recently been identified as essential for operation of the methylmalonyl pathway of propionate utilization in *M. tuberculosis* [47]. Both of these could potentially provide new targets for antituberculous drugs. Biotin is involved in many biosynthetic reactions, most notably fatty acid synthesis, where it is a cofactor for the acetyl-CoA carboxylase carboxytransferase function; so it was surprising to find that many genes involved in its synthesis are down-regulated in the macrophage. The result may indicate that the proposed remodeling of the cell envelope indicated by our analysis takes place without *de novo* synthesis of fatty acids, possibly by utilizing host-derived lipids as substrates; or the result could indicate increased lipid synthesis is mediated by post-transcriptional control not captured in this analysis. Metabolites involved in the synthesis of several aromatic amino acids were also found to be associated with up-regulated genes by DPA analysis. The results are consistent with the finding that many amino acid auxotroph of *M. tuberculosis* are attenuated *in vivo* indicating that the pathogen is unable to obtain these amino acids from the host [48] [49].

Concomitant with the DPA signal indicating increased cell wall lipid synthesis, the analysis indicated that metabolites involved in central metabolism were generally associated with down-regulated genes when *M. tuberculosis* enters the macrophage. This down-regulation of many (but not all) central metabolic pathways was also found for *in vitro* perturbations such as slow growth in the chemostat and exposure to hydrogen peroxide and is consistent with our previous study indicating that down-regulation of growth rate is likely to be a key early component of the adaptation of *M. tuberculosis* to the macrophage environment [18]. This finding is perhaps puzzling given the above indications of increased biosynthetic metabolism towards lipid and amino acid synthesis. Once again, the explanation may be that the early adaptation of the tubercle bacillus to the macrophage environment is achieved mostly by a redirection of metabolism using preexisting metabolites, rather than *de novo* synthesis of the those metabolites via the metabolic precursors generated from central metabolism: a remodeling rather than a renewal of the cell.

DPA of sputum-derived *M. tuberculosis* similarly revealed an up-regulation of metabolites involved in cell wall and virulence factor synthesis, with some similarities but also clear differences from analysis of the macrophage data. Metabolites associated with up-regulated genes from *M. tuberculosis* cells derived from human sputum (but not mouse macrophages) were involved in synthesis of the sulfolipid virulence factor SL-1 and also peptidoglycan synthesis; whereas the macrophage derived cells (but not sputum-derived cells) were associated with phenolic glycolipid (PGL) and acyltrehalose synthesis. The results indicate that mycobacteria isolated from human sputum have quite a different metabolic signature from mycobacteria grown in mouse macrophages. DPA of sputum down-regulated genes also identified several metabolites involved in molybdenum synthesis, a cofactor of nitrate reductase, *narX*, involved in anaerobic respiration. It is possible that this response indicates a switch away from anaerobic respiration in sputum-derived *M. tuberculosis* cells.

It is also notable that none of the *in vitro* conditions examined generated the same DPA metabolic profile as *in vivo*-grown *M. tuberculosis* cells indicating that there are aspects of *in vivo* metabolism of *M. tuberculosis* that are not captured by any single *in vitro* model. The analysis may thereby shed light differences between drug sensitivity *in vivo* and *in vitro*. For instance, isoniazid is widely used to prevent progression from latent to active disease [50] but has no effect on *M. tuberculosis* in the Wayne model of dormancy [51]. DPA provides insight into the underlying similarities and differences between the metabolic state of *M. tuberculosis in vitro* and *in vivo* that may provide clues to development of novel *in vitro* systems that more closely simulate the environmental conditions experienced by *M. tuberculosis in vivo*.

In summary, the method described here provides a novel way of using a metabolic network to analyze gene expression data. The method can be automatically applied to data to provide novel hypotheses concerning hidden metabolic signals. Potentially, DPA can be applied to other high-throughput data sets (e.g. proteomics) to integrate more information and capture other levels of control (e.g. protein degradation as opposed to mRNA degradation). Here we have demonstrated the utility of the method by using DPA to analyze the gene expression response of *M. tuberculosis* to the macrophage environment. The data suggests that the tubercle bacillus responds to the macrophage environment by shutting down central metabolic pathways but increasing activity in lipid biosynthetic pathways possibly in an effort to remodel its surface in anticipation of attack by host defenses. The analysis also demonstrated that the response of *M. tuberculosis* to the intracellular environment could be deconstructed into components that can be studied *in vitro*. The DPA method may be used to extract metabolic information from any microarray dataset where the appropriate metabolic model is available.

## Materials and Methods

The aim of DPA is to identify a set of metabolites whose production/consumption (producibility) is expected to be different between two conditions on the basis of gene expression differences. The method utilizes has some features in common with another method, Analysis of Differentially Affected Metabolites (ADAM) that will be described elsewhere (E. Laing *et al.*, paper in preparation). To perform DPA analysis we first utilize a FBA-based metabolite producibility plot to identify, at a system-level, the sets of genes that participate in the production of each metabolite. This is essentially a binary matrix that links genes with metabolites on the basis of whether or not each gene is essential for production of each metabolite. Subsequently, for each of the metabolites, we calculate a metabolite signal, defined as the median microarray-derived data signal for those genes that affect its production. Therefore, for each microarray dataset, representing the experimental condition of interest, we generate a vector of metabolite signals that we sometimes refer to as the *metabolite state*. These data may then be subjected to cluster analysis to identify experimental conditions that result in similar changes in global metabolite state (the sum of all metabolites in the cell) and metabolites that have common metabolite signal profiles across the experimental conditions examined. To avoid negation of gene expression signals for metabolites which were associated with different sets of up- and down-regulated genes, the analysis was performed separately for up and down-regulated genes. Each metabolite in the network was then ranked according to the average intensity of microarray signal associated with genes that affect its production (Figure 1, step 3) using the non-parametric Rank Products Analysis [32], which has been shown to be the method of choice for meta-analysis of microarray datasets derived by different research groups on different experimental platforms. Rank Product Analysis has been shown to be robust against experimental noise and performs well in comparison to other typical microarray analysis strategies [52]. The workflow of DPA analysis is illustrated in Figure 1; below we describe in detail the individual steps.

### Flux balance analysis

We used Flux Balance Analysis (FBA) to interrogate our genome scale model of *M. tuberculosis* metabolism to obtain lists of genes associated with the synthesis of metabolites in the network. FBA has become a standard method for large-scale metabolic network modeling and is discussed in detail in numerous articles [53]–[55]. In this work we calculate the maximal theoretical flux towards each metabolite in the network. Following definition of the metabolite producibility in [56] the row of the stoichiometric matrix corresponding to the metabolite in question is used to define the objective function. Note that exactly the same value of metabolite producibility is obtained if a new reaction that consumes the metabolite as its only substrate is introduced to the model and the flux through this reaction is used as the objective function. The GSMN-TB model provides Boolean formulas describing association between genes and reactions. These formulas are used to identify reactions, which require activity of each gene. A detailed description of the FBA calculations can be also found in our previous work, which describes the development of the GSMN-TB model and accounts for assumptions specific to this model such as “replenishing flux” used to model requirement for enzyme cofactors. All FBA calculations were as described in [18].

### Producibility plot

In the first step of DPA, for each metabolite, a set of genes that are required for the reactions affecting the cellular level of the metabolite is obtained. Our aim was to not only take into account the genes participating in reactions synthesizing or consuming each metabolite, but to also consider global effects *i.e.* the existence of reactions anywhere in the network that affects the flux towards that metabolite. For this analysis we utilized the producibility plot method formulated by Imielinski [56], making each metabolite *m* an objective function of a single Flux Balance Analysis optimization and iterate the procedure over all metabolites in the network. For the analysis of *M. tuberculosis*, to capture gene-metabolite associations under a variety of conditions, the FBA was performed separately with the substrates glucose, glycerol, acetate, propanoate and cholesterol and the resulting associations pooled. The maximal theoretical flux towards metabolite m in a wild-type model in which all genes are active was first calculated. Subsequently, we calculate the maximal theoretical flux towards metabolite m in the network in which each reaction in the model annotated as catalyzed by product of gene g is inactivated, i.e. the fluxes of all reactions, which involve g are constrained to 0. For each pair *m*, *g* we then calculate a difference ΔF_m_,*_g_* = F_m_,*_g_*−F_m_,_wt_, between the maximal flux towards *m* in the gene *g* knockout model (F_m_,*_g_*) and the wild type model (F_m_,_wt_). This results in a matrix P, in which rows are indexed with metabolites, columns are indexed with genes and which cells contain ΔF_m,g_ values for all gene-metabolite pairs. We represent P in binary form in which we assign 1 to all ΔF_m,g_ values different from 0 (0.001 absolute value cut-off is used) and assign 0 to the remaining cells in the matrix, where no change is observed as the result of *g* inactivation. The binary form of matrix P can be visualized as a dot-plot and is referred to as the Producibility Plot. In the remaining part of this article we will also refer to the maximal flux towards metabolite *m* as the *producibility* of *m*.

The resulting metabolite producibility is a function of the “structural network” and reaction reversibility (and hence reaction bounds), or simply, topology and physical constraints of the network.

### Calculation of metabolite signals from microarray data

In the second step of DPA we incorporate data from the microarray datasets representing different experimental conditions. The log_2_ ratios of treatment and reference sample signals as calculated by authors of these datasets were used (no processing of data was conducted); genes were classed as up-regulated if they had positive log_2_ signal ratios and down-regulated if negative.

To analyse microarray data in the context of the GSMN-TB model we define a ‘**metabolite signal**’ as the median log_2_ microarray signal ratio of genes influencing producibility of the given metabolite. To calculate metabolite signals we first use the producibility plot to identify for each metabolite *m* in the network a set *S_m_* of genes that affect its producibility. These genes are identified from a set of non-zero elements in the rows of the binary producibility plot corresponding to *m*. To integrate data from array *a*, for each metabolite we calculate the median up-regulated metabolite signal, *U_m,a_* , from all up-regulated genes in array *a* in S*_m_*. Similarly, all down-regulated genes in S*_m_* were used to calculate the metabolite signal of downregulated genes D_m,a_. As a result, we obtain metabolite signal matrices U and D, where rows are metabolites, columns arrays, and values are metabolite signals due to the up and down regulated genes respectively. It is these matrices upon which all subsequent clustering and Rank Products analysis are based.

### Cluster analysis

We performed hierarchical cluster analysis (HCL) to identify groups of experimental conditions that induce similar metabolic responses, as identified by DPA, in the *M. tuberculosis* bacillus. Here we ranked all metabolite signal values in each column of the U and D metabolite signal matrices, resulting in matrices U*_ranked_* and D_ranked_. Thus the row for each metabolite *m* in U*_ranked_* and D_ranked_ contains the ranks of the metabolite signal of *m* across all treatment/reference microarrays. Subsequently, the hierarchical clustering was performed with the pvclust package of the R environment, with Pearson correlation as a distance measure and complete linkage [57]. The pvclust package was also used to calculate statistical significance of clusters by the bootstrap method [57] for assessing the uncertainty in hierarchical cluster analysis, 10,000 bootstrap replicates were generated. Tree nodes, which were retained in 90% of the replicates, as calculated by the Approximately Unbiased (au) multiscale bootstrap method [57], were considered statistically significant.

### Rank products analysis

The Rank Product analysis is a non-parametric statistical test, which has been developed for identification of differentially expressed genes in microarray data [32]. Briefly, the method analyses a data table where rows represent cases (genes) and columns represent repeated measurements. In the one-class case the columns of the data table contain signal log ratios for the comparison of two conditions. In a two class-case the columns are assigned to two classes and the software calculates all pairwise differences between the log signal ratios of columns belonging to the two classes. In the next step the program ranks each column of log-signal ratios and identifies cases (genes), which consistently rank at top of the list in each repetition of the experiment. For each case the product of ranks of this case in each of the replicates is calculated. This obtained value is compared to the values calculated in multiple randomized datasets (shuffling of case names) to calculate P-values and corresponding Percentage of False Discovery. Because the method is based on ranks it is robust with respect to many assumptions, such as normality and is particularly suitable to meta-analysis of datasets collected in different laboratories and on different experimental platforms.

In our work we used Rank Products analysis to identify differentially produced metabolites. The U and D matrices described above were submitted to the RankProd function of the Bioconductor package in the R environment. By using all treatment/reference log_2_ ratios as input to one-class Rank Products analysis (a method typically applied to two-colour direct comparison microarray experiments) we feed all the replicates of the experiments as a data column (if no replicate available then treat every experimental condition as a ‘replicate’); for any metabolite to be identified as being significantly different between the ratio numerator and denominator there must be consistency across experimental conditions. Thus, this analysis identifies common features of *E. coli* and *M. tuberculosis* metabolic response to different experimental conditions. To identify differences between metabolite states *between* experimental conditions a two-class Rank Products analysis of multiple treatment/reference ratios for each condition was employed. The P-value and PFP (probability of false prediction) value obtained from the output of Rank Products analysis was used to determine the significance of the metabolic response.

To check the robustness, we examined the DPA based rank product analysis in *M. tuberculosis* and *E. coli* datasets.

### Datasets and metabolic model

For *E. coli* DPA we used the *iAF1260* genome scale metabolic network [33]. All the FBA simulation of producibility plot for *E. coli* were performed in pyFBA (Python based unpublished toolbox) with the GLPK linear programming toolkit. The *E. coli* transcriptome database that was analyzed was taken from a microarray study of the *E. coli* growing in aerobic and anaerobic conditions, both wild-type and an FNR- strain [25].

DPA was also applied to transcriptome data sets representing the global gene expression program of the *M. tuberculosis* bacillus in the macrophage environment, human sputum and a range of *in vitro* environmental perturbations. These data were analyzed in the context of the previously published GSMN-TB genome-scale metabolic network [18]. Transcriptome data sets and the GSMN-TB model are described below.

The gene expression data obtained from Schnappinger [22], is from *M. tuberculosis* cells harvested at 4, 24 and 48 hours after infection of macrophages. Each dataset contains the result of a ‘two-color’ microarray hybridization experiment with the reference condition in each case being bacteria grown to mid-log phase in 7H9, a standard *M. tuberculosis* culture medium. We chose the activated macrophage 48 hr after infection (referred to as macrophage) data as a representative of the *in vivo* state of the TB bacillus. The *in vitro* conditions from the same experimental dataset included experiments where the TB bacillus was exposed to 5 mM hydrogen peroxide for 40 minutes (peroxide.1) and cells grown on palmitic acid as a carbon source (palmitate.1). Other *in vitro* datasets were taken from a large study of *M. tuberculosis* exposed to a variety of environments [58]. We chose the following datasets: Growth on succinate (succinate), oxygen-limited NRP-1 cells which represent cells grown in the ‘Wayne model’ of *M. tuberculosis* dormancy [38], starvation, pH 4.8, UV radiation, NaN3 treatment (azide), hydrogen peroxide treatment (peroxide.2) and growth on palmitate (palmitate.2). These *in vitro* conditions were chosen to model aspects of the presumed *in vivo* environment of the TB bacillus. The third dataset was taken from the study of [37] of human sputum-derived *M. tuberculosis* samples. Finally, we also analyzed a transcriptional dataset obtained from our own studies that represents the response of *M. bovis* BCG to slow growth in the chemostat with glycerol as a sole carbon source [44]. All of the microarray datasets were converted to log_2_ fold change (treatment/reference) ratios for ease of comparison. Table 1 shows the abbreviations used for each experimental condition and the data source.

The previously published genome scale metabolic network of *M. tuberculosis* [18] was modeled using flux balance analysis, as described. An updated GSMN-TB model can be obtained in the tab delimited text or SBML format from the website http://sysbio3.fhms.surrey.ac.uk/.

## Supporting Information

Figure S1 DPA of all *M. tuberculosis* experimental datasets displayed as a heat map with metabolite rank displayed as intensity of color. DPA of up-regulated genes is shown on the left in red and DPA of down-regulated genes is displayed on the right in green colour. Clustering of experiments is indicated as a dendrogram at the top of each heat map and clustering of metabolites is shown on the left.
(TIFF)

Table S1 Metabolites associated with up- and down-regulated genes in *E. coli*. Table S1a, metabolites identified as associated with down-regulated genes on exposure of wild-type *E. coli* to anaerobic conditions (corresponding to regulatory patterns II, IV, V of [25]), Table S1b, metabolites associated with up-regulated genes in the wild-type in response to anaerobic growth (regulatory patterns I, III, VII), Table S1c, metabolites associated with increased gene expression in the FNR- strain grown under anaerobic conditions (regulatory patterns I, V, VI), and Table S1d, metabolites associated with decreased gene expression in the FNR-strain grown under anaerobic conditions (regulatory patterns II, VII, VIII).
(XLS)

Table S2 Top 100 metabolites predicted by DPA and identified by one-class Rank Products analysis (see Materials and Methods) as associated with up-regulated and down-regulated *M. tuberculosis* genes for each dataset.
(XLS)

Table S3 Metabolites that are shared between different experimental conditions (Venn diagram, Figure 4) associated with up-regulated *M. tuberculosis* genes.
(XLS)

Table S4 Metabolites that are shared between different experimental conditions (Venn diagram, Figure 4) associated with down-regulated *M. tuberculosis* genes.
(XLS)

Table S5 Metabolite assignments classified into broad areas of metabolism based on the pathway(s) that involved each metabolite in the GSMT-TB genome-scale metabolic network model.
(XLS)
