# Supplementary figures and images for: Differential Producibility Analysis (DPA) of Transcriptomic Data with Metabolic Networks: Deconstructing the Metabolic Response of M. tuberculosis
Source: PLoS Comput Biol. 2011 Jun 30;7(6):e1002060. doi: 10.1371/journal.pcbi.1002060 (PMC3127818; doi:10.1371/journal.pcbi.1002060)

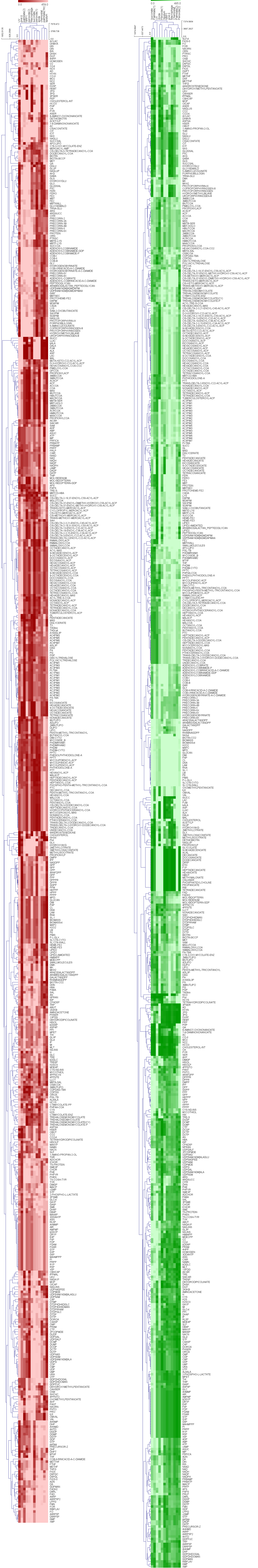

Supplement: Figure S1 — DPA of all M. tuberculosis experimental datasets displayed as a heat map with metabolite rank displayed as intensity of color. DPA of up-regulated genes is shown on the left in red and DPA of down-regulated genes is displayed on the right in green colour. Clustering of experiments is indicated as a dendrogram at the top of each heat map and clustering of metabolites is shown on the left. (TIFF) [file pcbi.1002060.s001.tiff]
